# Evidence for changing lipid management strategy to focus on non-high density lipoprotein cholesterol

**DOI:** 10.1186/s12944-019-1080-x

**Published:** 2019-06-07

**Authors:** Xin Su, Yi Kong, Daoquan Peng

**Affiliations:** 1 Department of Cardiovascular Medicine, the Second Xiangya Hospital of Central South University, No. 139 Middle Renmin Road, Changsha, 410011 Hunan China; 2 Department of Dermatology, Hunan Key Laboratory of Medical Epigenomics, the Second Xiangya Hospital, Central South University, Changsha, Hunan China

**Keywords:** LDL-C, Non-HDL-C, Goals, Coronary heart disease, Risk

## Abstract

Low-density lipoprotein cholesterol (LDL-C) has been recommended as the primary treatment target on lipid management in coronary heart disease (CHD) patients for past several decades. However, even by aggressive LDL-C lowering treatment, patients still present a significant residual risk of major adverse cardiovascular events (MACE). Non-high-density lipoprotein cholesterol (non-HDL-C) contained all the atherogenic lipoproteins, such as chylomicron, very-low density lipoprotein (VLDL), LDL, intermediate density lipoprotein (IDL). Many prospective observation studies have found that non-HDL-C was better than LDL-C in predicting risks of MACE. Since non-HDL-C appears to be superior for risk prediction beyond LDL-C, current guidelines have emphasize the importance of non-HDL-C for guiding cardiovascular prevention strategies and have flagged non-HDL-C as a co-primary therapeutic target. The goals of non-HDL-C were recommended as 30 mg/dl higher than the corresponding LDL-C goals, but the value seemed inappropriate. This review provide evidence for changing lipid management strategy to focus on non-HDL-C and appropriate values for adding to LDL-C goals would be proposed.

## Introduction

The prevalence of coronary heart disease (CHD) in both developed and developing countries has risen markedly, posing serious risks to future health of humans and leading to a high mortality [1–3]. Nowadays, the relationship between hypercholesterolemia and CHD has been well established [4–6], and lipid-lowering therapy is an important strategy in primary and secondary prevention of CHD [7, 8]. In the past few decades, low-density lipoprotein cholesterol (LDL-C) was recommended as the primary treatment target on lipid management in CHD patients [9–11]. Historically, when it comes to decreasing CHD risk, most lifestyle and pharmacologic interventions focused on reducing LDL-C. Selected worldwide dyslipidemia guidelines and expert recommendations, including the American Diabetes Association/American College of Cardiology (ADA/ACC) guidelines [12], Canadian Cardiovascular Society (CCS) guidelines [13] and European Society of Cardiology/European Atherosclerosis Society (ESC/EAS) guidelines [14], have all identified LDL-C targets of < 70 and < 100 mg/dl for patients at very high- and high-risk for CHD, respectively, and recommended the first-line therapy should be directed toward LDL-C lowering.

**Fig. 1:**
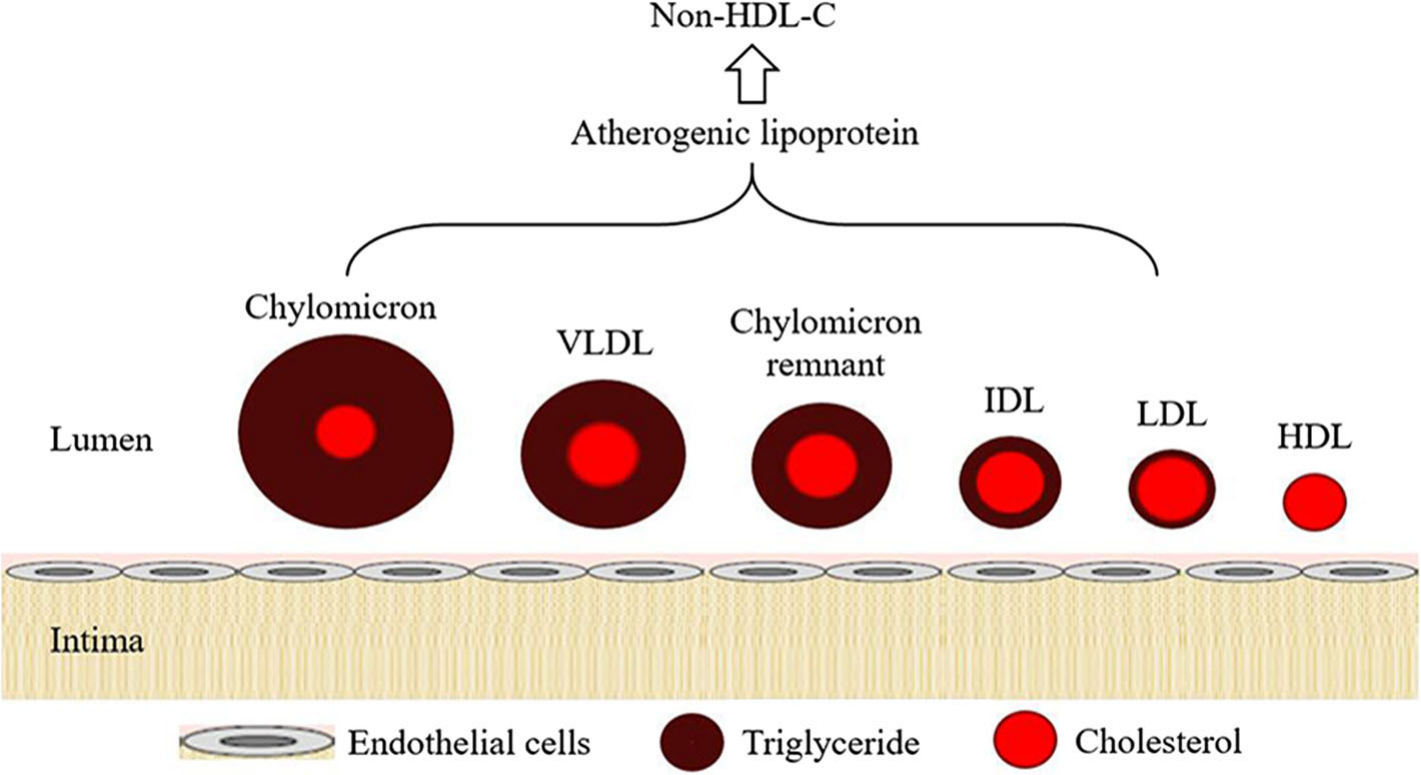
Important lipids in human plasma. Non-HDL-C, calculated as [total cholesterol – (HDL-C)], quantifies all atherogenic apolipoprotein B-containing lipoproteins, including LDL, very low-density lipoprotein (VLDL), intermediate-density lipoprotein (IDL), chylomicrons, and their TG-rich lipoprotein remnants

In contrast to LDL-C, non-high-density lipoprotein cholesterol (non-HDL-C) receives much less attention and remains as a co-primary therapeutic target associated with LDL-C or as a secondary target when triglyceride (TG) > 200 mg/dl within current guidelines [15, 16]. Indeed, non-HDL-C quantifies all atherogenic apolipoprotein B-containing lipoproteins, including LDL, very low-density lipoprotein (VLDL), intermediate-density lipoprotein (IDL), chylomicrons (CM), and their TG-rich lipoprotein remnants (Fig. 1), whose contribution to atherogenic risk is accounted for by non-HDL-C but not LDL-C alone [17]. Additionally, non-HDL-C is simply calculated by subtracting HDL-C from total cholesterol (TC) [18] and is not influenced by fasting conditions, which could provide convenience for patients [19]. For these reasons, the significance of non-HDL-C in predicting CHD risks is being well recognized. Currently, several important guidelines, including International Atherosclerosis Society (IAS) guideline [20], National Lipid Association (NLA) guideline and National Institute for Health and Care Excellence (NICE) guideline [21], have flagged non-HDL-C as a primary therapeutic target for patients with CHD. However, whether non-HDL-C or LDL-C is the better marker reflecting the atherogenic coronary risk remains controversial.

## Residual risk of CHD beyond LDL-C after using lipid-lowering therapy

It is no doubt about the relationship between plasma levels of LDL-C and risks of CHD, as well as about the benefits of lipid-lowering therapy such as statin treatment. Over the past two decades, several large-scale randomized controlled trials, including the Scandinavian Simvastatin Survival Study (4S) [22, 23], the Long-Term Intervention with Pravastatin in Ischemic Disease (LIPID) study [24, 25], the Justification for the Use of Statins in Prevention: an Intervention Trial Evaluating Rosuvastatin (JUPITER) study [26, 27], have demonstrated the beneficial effects of statins in lowering the risk of CHD. As such, lipid-lowering agents have become a mainstay of therapy in the primary and secondary prevention of major adverse cardiovascular events (MACE) [28–31]. However, despite the success of LDL-C lowering, it is also clear from the evidence that the persistence of a high CHD risk, as a concept called residual risk, are notable after using lipid-lowering agents [32–34].

There is evidence that even achieving lower LDL-C targets (< 70 mg/dl) recommended by current guidelines in very high-risk patients may still leave a high residual risk of MACE. In the 4S trial, patients treated with statin therapy experienced CHD event rates approximating 19% (compared to 28% with placebo) over the 5-year study period [35, 36]; in the Cholesterol and Recurrent Events (CARE) trials, plasma LDL-C of patients with 40 mg pravastatin each evening reduced during follow-up by 28%, but the residual risk of MACE remained 10.2% compared to 13.2% in patients with placebo [37, 38]. Similar results were observed in other studies such as the Heart Protection Study (HPS) [39–41]. Additionally, a meta-analysis including 90,056 individuals from 14 randomized trials reported that statin therapy reduced the risk of major vascular events by 23% for each 1 mmol/L LDL-C lowering. However, the residual risk of MACE over a 5-year period remained high; 14% of patients experienced a cardiovascular event despite being allocated to the statin group, compared with 18% among patients allocated to placebo [42].

The subsequent analyses evaluated the effects of high-dose statin treatment for more intensive LDL-C lowering. In the Treating to New Targets (TNT) trial, where treatment with atorvastatin 80 mg daily was associated with a 22% relative risk reduction of MACE compared with treatment with atorvastatin 10 mg daily, one in 11 patients experienced a cardiovascular event during 5-years follow-up; in the Pravastatin or Atorvastatin Evaluation and Infarction Therapy-Thrombolysis In Myocardial Infarction 22 (PROVE IT-TIMI22) and the Incremental Decrease in Endpoint through Aggressive Lipid Lowering (IDEAL) trials, high-intensity (80 mg atorvastatin daily) therapy demonstrated greater CVD risk reduction when compared to moderate-intensity statin therapy, but residual risk in the intensive treatment arms was still 22.4 and 12%, respectively, despite mean LDL-C levels that were not elevated [43–45]. Since the persistently residual CHD risk despite high-intensity statin therapy, the consideration of adjunctive therapies has been prompted to reduce further risk of CHD. Indeed, the IMProved Reduction of Outcomes: Vytorin Efficacy International Trial (IMPROVE-IT) was designed to assess the benefits of ezetimibe to moderate-intensity simvastatin. Once again, the rate of MACE decreased significantly, but patients in the treatment by lipid-lowering agents still presented a 32.7% residual risk (compared to 34.7% with simvastatin alone) [46–48].

More recently, Nichols et al. determine whether high TG in the presence of statin-controlled LDL-C influence the risk of CHD among patients with diabetes by using the data about adults with diabetes from the Southern California and Pacific Northwest regions of Kaiser Permanente. The incidence rate for non-fatal MI was 30% higher in the high TG group [Rate Ratio (RR) =1.30; 95% CI, 1.08~1.58]. The rate was 23% higher for non-fatal stroke (1.23, 1.01~1.49), 21% higher for coronary revascularization (RR = 1.21; 95% CI, 1.02~1.43) and 33% higher for unstable angina (RR = 1.33; 95% CI, 0.87~2.03) [49]. We can infer from these results that TG levels might lead to the excess risk of MACE in patients with high level of TG. Thus, even the LDL-C level was controlled by lipid-lowering agents, rate of MACE was greater among patients with diabetes and high TG levels.

## Non-HDL-C as a better predictor for CHD

Almost all currently available guidelines have stressed that LDL-C levels should be used as the primary target to lipid-lowering therapy. However, trials about the efficacy of lipid-lowering therapy have shown that the cardiovascular benefits of statins may go beyond their influence on LDL-C levels. Thus, LDL-C may not be the best lipid parameter to predict cardiovascular risk or to quantify the athero-protective effect of lipid-lowering agents.

A number of studies have investigated the relationships between LDL-C or non-HDL-C and the risk of CHD in the past three decades. Early in 2001, data from the Lipid Research Clinics Program Follow-up Study, designed to determine whether non-HDL-C could be useful in predicting CHD mortality over a 19-year follow-up in 2406 men and 2056 women, revealed that levels of non-HDL-C at baseline was significant and strong predictors of CHD deaths while LDL-C level was a somewhat weaker predictor in both sexes. Differences of 30 mg/dl in non-HDL-C and LDL-C levels corresponded to increases in CHD risk of 19, 15% in men and 11, 8% in women, respectively. Compared with men with non-HDL-C < 160 mg/dl, those with non-HDL-C > 220 mg/dl had a hazard ratio (HR) for future CHD of 2.14 (95% CI, 2.50~3.04). Compared with men with LDL-C > 130 mg/dl, men with LDL-C > 190 mg/dl had a HR for future CHD of 1.77 (95% CI, 1.22~2.59). Results were similar among women [50].

A nested case-control study among 18,225 participants of the Health Professionals Follow-up Study was designed to compare non-HDL-C and LDL-C as predictors of CHD. After adjustment for matching factors, the relative risk of CHD in the highest quintile compared with the lowest quintile was 2.76 (95% CI, 1.66~4.58) for non-HDL-C and 1.81 (95% CI, 1.12~2.93) for LDL-C. When non-HDL-C and LDL-C were mutually adjusted, only non-HDL-C was predictive of CHD, suggesting that non-HDL-C could be more strongly associated with CHD risk than LDL-C [51]. Similarly, data from the Framingham Heart Study (2693 men and 3101 women) and showed that on the basis of the joint distributions of LDL-C and non-HDL-C, after multivariate adjustment, no association was found between LDL-C and the risk for CHD within non-HDL-C level; whereas within LDL-C levels, a strong positive and graded association between non-HDL-C and risk for CHD was observed [52].

Recently, a meta-analysis including 8 statin trials found that patients with on treatment LDL-C < 100 mg/dl but non-HDL-C > 130 mg/dl had significant a HR for future CHD of 1.32 (95% CI, 1.17~1.50) compared to those reaching both targets. In contrast, patients with on treatment LDL-C > 100 mg/dl but non-HDL-C < 130 mg/dl had similar HR for future CHD of 1.02 (95% CI, 0.92~1.12) as those reaching both targets [53]. In 2013, in the European Prospective Investigation into Cancer and Nutrition (EPIC)-Norfolk prospective population study containing 25,639 men and women aged 45~79 years, researchers found that the multivariable-adjusted HR of future CHD was 1.22 (95% CI, 1.17~1.27) for LDL-C and 1·26 (95% CI, 1.20~1.31) for non-HDL-C, respectively. The multivariable-adjusted HR of future CHD in the highest quartile was 1.67 (95% CI, 1.47~1.91) in LDL-C and 1.87 (95% CI, 1.62~2.15) in non-HDL-C [54].

Furthermore, in 2016, Zhang et al. studied 1757 consecutive subjects undergoing coronary angiography and found non-HDL-C (HR = 1.33; 95% CI, 1.16~1.51) was slightly superior to LDL-C (HR = 1.28; 95% CI, 1.13~1.46) in predicting high severity of CHD after adjusting for potential confounders. In 2017, a retrospective study investigated the predictability of attaining non-HDL-C goal and long-term MACE in Thai patients after acute myocardial infarction (AMI) compared to attaining LDL-C target. During mean follow-up of 2.6 years among 868 patients after AMI, 34.4% achieved non-HDL-C target, 23.7% achieved LDL-C target and 21.2% experienced MACEs. Compared to those with non-HDL-C level less than 100 mg/dl, patients with non-HDL-C greater than 130 mg/dl had a HR for MACE of 3.15 (95% CI, 1.46~6.80). However, compared to those with LDL-C level less than 70 mg/dl, patients with LDL-C greater than 100 mg/dl was associated with reduced risk of MACE (HR = 0.42; 95% CI, 0.18~0.98) after direct pairwise comparison with non-HDL-C level. These results indicated that non-HDL-C was a better predictor of future MACE following AMI [55]. Another study in 2017 indicated that non-HDL-C was good predictors of the risk of increased arterial stiffness in postmenopausal women in an urban Brazilian population [56]. Since increased arterial stiffness is an independent risk of CHD, this result also provided the evidence that non-HDL-C could better reflect the risk of CHD. In a large cohort analysis about among 62,428 statin-treated individuals with type 2 diabetes mellitus in healthcare delivery system of Kaiser Permanente Northern California, the relevance of non-HDL-C goals for primary prevention of CHD among patients with diabetes was assessed. After adjusted, the risk of incident CHD for these statin-treated patients was lower with decreasing achieved non-HDL-C levels (*P* < 0.001). Relative to achieved non-HDL-C ≥ 160 mg/dl, non-HDL-C < 80 mg/dl had HR = 0.59 (95% CI, 0.51~0.68). [57]. In a total of 13 studies with 156,381 individuals, the pooled RR of CHD was 1.59 (95% CI, 1.46~1.72) in the general population and 1.99 (95% CI, 1.57~2.51) in type 2 diabetes patients. Subgroup analysis showed the similar effect of non-HDL-C on CHD risk between men (RR 1.98; 95% CI, 1.70~2.30) and women (RR 1.63; 95% CI, 1.35~1.96) [58]. These findings support the use of non-HDL-C treatment goals for CHD primary prevention in diabetic patients. In a word, non-HDL-C was better than LDL-C in the prediction of future MACE.

## Non-HDL-C as a better target of lipid-lowering therapy

As mentioned above, to address therapeutic adequacy, certain worldwide guidelines have designated secondary targets for non-HDL-C (< 100 and < 130 mg/dl) when LDL-C goal have been achieved in very high- risk and high-risk patients, respectively, with TG levels between 200~500 mg/dl. Evidence showed that the agents for lipid-lowering therapy to reduce cardiovascular risk, such as statins, also lower non-HDL-C. In 2008, a post-hoc analysis, combined data from 2 prospective and randomized clinical trials in which 10,001 patients from the TNT trial and 8888 patients from the IDEAL trial with established CHD with usual-dose or high-dose statin treatment, showed that in models with LDL-C and non-HDL-C were positively associated with cardiovascular outcomes, whereas a positive relationship with LDL-C was lost. When LDL-C and non-HDL-C were included simultaneously, the positive relationship between LDL-C and major cardiovascular events was lost, whereas non-HDL-C retained its positive association with the occurrence of such events (HR = 1.31; 95% CI, 1.19~1.44) [59]. These results indicated that in patients with lipid-lowering treatments, level of non-HDL-C was more closely associated with cardiovascular outcome than level of LDL-C. In addition, to deeply explore the relationship between non-HDL-C reduction and CHD risk reduction for various lipid-modifying therapies, Robinson et al. performed a meta-analysis and showed that for statins, each 1% decrease in non-HDL-C resulted in an estimated 4.5-year CHD relative risk of 0.99 (95% CI, 0.98~1.00), and similar results were shown by using other lipid-lowering agents such fibrate and niacin. Most lipid-modifying drugs used as monotherapy have a one to one relationship between percentage non-HDL-C lowering and CHD reduction [60].

More recently, data from 1792 individuals who underwent percutaneous coronary intervention (PCI) from January 2004 to December 2009 were analyzed by Lee. All LDL-C and non-HDL-C variability parameters were independent predictors for MACE after adjusting for potential confounding factors. The authors demonstrated that 1 standard deviation (SD) increase corrected variability independent of mean (cVIM) of LDL-C and non-HDL-C increased the risk of MACE by 34% (HR = 1.34; 95% CI, 1.18~1.52) and 37% (HR = 1.37; 95% CI, 1.20~1.57), respectively [61]. Another analysis from Tehran lipid and glucose study including 5474 participants showed that during a median follow-up of 8.9 years and after adjustment, each 1-SD increase in LDL-C and non-HDL-C was associated with 12% (HR = 1.15; 95% CI, 1.05~1.21) and 16% (HR = 1.38; 95% CI, 1.25~1.46) risk for T2DM, respectively (*P* < 0.05) [62]. These findings indicated that non-HDL-C treatment goals could be a better target of lipid-lowering therapy.

## Recommended value of non-HDL-C goals

Non-HDL-C has been verified as an important target of therapy for CHD [63–65]. However, the goal of non-HDL-C still remains ambiguous. Current recommendations set the goal of non-HDL-C as 30 mg/dl higher than the corresponding LDL-C goals [66–68]. That is, a patient with a LDL-C goal of 70 mg/dl would have a corresponding non-HDL-C goal of 100 mg/dl. As mentioned above, non-HDL-C, calculated as the difference between TC and HDL-C, represents the cholesterol mass contained in all atherogenic lipoproteins including LDL-C and VLDL-C. The rationale for difference of 30 mg/dl between LDL-C and non-HDL-C goals was based on the assumption that VLDL-C was the principal atherogenic lipoprotein after LDL-C [69]. It is proposed that, on the average, the weight ratio of TG to cholesterol in VLDL particle is 5 to 1 [70]; that is, if the weight of TG is 150 mg in VLDL particle, the weight of cholesterol content in VLDL particle should be around 30 mg. Recent evidence suggested that a biologically optimal fasting TG level was less than 150 mg/dl [71–73], so a normal VLDL-C level was likely less than 30 mg/dl.

Actually, evidence from the Limiting UNdertreatment of lipids in ACS With Rosuvastatin (LUNAR) Trial have already shown that to better match LDL-C, the current non-HDL-C goal should be lowered by 8~10 mg/dl [74]. In 2013, an analysis revealed that LDL-C cut-points of 100, 130, 160, and 190 mg/dl were in the same population percentiles as non-HDL-C values of 125, 157, 190, and 223 mg/dl in 1,310,440 U.S. adults population [75]. In 2014, Kuwabara et al. used baseline cross-sectional data of 4110 participants from two studies: the KOBE Study and the Tsuruoka Metabolomic Cohort Study and evaluated whether the difference between LDL-C and non-HDL-C in the general Japanese population. They found the mean difference between the non-HDL-C and LDL-C was 19.6 mg/dl and 24.1 mg/dl for men for the KOBE Study and Tsuruoka Metabolomic Cohort Study, respectively [76]. Similar difference was also observed in women. In both cohort studies, the difference was lower than 30 mg/dl. More recently in 2018, Brito et al. investigated 14,837 participants from the Longitudinal Study of Adult Health (ELSA-Brasil) and also found obvious difference and discordance in Brazilian population as the non-HDL-C values, based on correspondent LDL-C population percentiles (70, 100, 130, 160, and 190 mg/dl), were 92, 122, 156, 191, and 223 mg/dl [77].

The discordance and difference between non-HDL-C and LDL-C provided the evidence that adding of 30 mg/dl to LDL-C goal as non-HDL-C goal seemed inappropriate and might over-estimate goal-reaching rate. Lowering 5~10 mg/dl of conventional non-HDL-C cut-points may better match percentiles of LDL-C cut-points. However, large-scale prospective studies in CHD patients are needed to determine the validity of the values.

## Conclusions

There is significant residual risk of MACE despite aggressive lipid-lowering therapy. Accumulating evidence support that the relationship between non-HDL-C lowering and reduction of cardiovascular risk. Non-HDL-C is a more comprehensive measure of atherogenic particles than LDL-C and is superior to LDL-C in its ability to predict MACE, thus, non-HDL-C has been shown to predict CHD similarly to apolipoprotein B. We suggest that in clinical practice, more attention should be directed to non-HDL-C and to achieving non-HDL-C goals in patients at increased cardiovascular risks. In summary, given the superiority of non-HDL-C in cardiovascular risk prediction beyond LDL-C, and the proven benefit of non-HDL-C lowering, future guidelines should emphasize the importance of non-HDL-C for guiding cardiovascular prevention strategies.

## Data Availability

Not applicable.

## Abbreviations

4S: The Scandinavian Simvastatin Survival Study
ADA/ACC: American Diabetes Association/American College of Cardiology
AMI: Acute myocardial infarction
CARE: The Cholesterol and Recurrent Events trials
CHD: Coronary heart disease
CI: Confidence interval
CM: Chylomicrons
ESC/EAS: European Society of Cardiology/European Atherosclerosis Society
HPS: The Heart Protection Study
IAS: International Atherosclerosis Society
IDEAL: The Incremental Decrease in Endpoint through Aggressive Lipid Lowering
IDL: Intermediate-density lipoprotein
IMPROVE-IT: The IMProved Reduction of Outcomes: Vytorin Efficacy International Trial
JUPITER: The Justification for the Use of Statins in Prevention: an Intervention Trial Evaluating Rosuvastatin study
LDL-C: Low density lipoprotein cholesterol
LIPID: The Long-Term Intervention with Pravastatin in Ischemic Disease study
MACE: Major adverse cardiovascular events
NICE: National Institute for Health and Care Excellence
NLA: National Lipid Association
Non-HDL-C: Non-high density lipoprotein cholesterol
PROVE IT-TIMI: The Pravastatin or Atorvastatin Evaluation and Infarction Therapy-Thrombolysis In Myocardial Infarction
RR: Rate Ratio
TC: Total cholesterol
TG: Triglyceride
TNT: The Treating to New Targets trial
VLDL: Very low-density lipoprotein

## References

[1] Zhang XH, Lu ZL, Liu L (2008). Coronary heart disease in China. Heart.

[2] Zhang Y, Wu NQ, Li S, Zhu CG, Guo YL, Qing P, Gao Y, Li XL, Liu G, Dong Q, Li JJ (2016). Non-HDL-C is a better predictor for the severity of coronary atherosclerosis compared with LDL-C. Heart Lung and Circulation.

[3] Zhou MG, Wang HD, Zhu J, Chen WQ, Wang LH, Liu SW, Li YC, Wang LJ, Liu YN, Yin P (2016). Cause-specific mortality for 240 causes in China during 1990-2013: a systematic subnational analysis for the global burden of disease study 2013. Lancet.

[4] Hovingh GK, Davidson MH, Kastelein JJP, O'Connor AM (2013). Diagnosis and treatment of familial hypercholesterolaemia. Eur Heart J.

[5] Raal FJ, Hovingh GK, Catapano AL (2018). Familial hypercholesterolemia treatments: guidelines and new therapies. Atherosclerosis.

[6] Stein EA (2002). Management of dyslipidemia in the high-risk patient. Am Heart J.

[7] Feldman HA, Zuber K, Davis J (2017). Dyslipidemia how low should we go? A review of current lipid guidelines. Physician Assistant Clinics.

[8] Stroes E (2005). Statins and LDL-cholesterol lowering: an overview. Curr Med Res Opin.

[9] Dembowski E, Davidson MH (2009). A review of lipid management in primary and secondary prevention. J Cardiopulm Rehabil Prev.

[10] Shimizu R, Torii H, Yasuda D, Hiraoka Y, Kitada N, Hashida T, Yoshimoto A, Kita T, Kume N (2015). Serum lipid goal attainment in chronic kidney disease (CKD) patients under the Japan atherosclerosis society (JAS) 2012 guidelines. J Atheroscler Thromb.

[11] Shirai K (2004). Obesity as the core of the metabolic syndrome and the management of coronary heart disease. Curr Med Res Opin.

[12] Stone NJ, Robinson JG, Lichtenstein AH, Merz CNB, Blum CB, Eckel RH, Goldberg AC, Gordon D, Levy D, Lloyd-Jones DM (2014). 2013 ACC/AHA guideline on the treatment of blood cholesterol to reduce atherosclerotic cardiovascular risk in adults A Report of the American College of Cardiology/American Heart Association Task Force on Practice Guidelines. Journal of the American College of Cardiology.

[13] Anderson TJ, Gregoire J, Pearson GJ, Barry AR, Couture P, Dawes M, Francis GA, Genest J, Grover S, Gupta M (2016). 2016 Canadian cardiovascular society guidelines for the Management of Dyslipidemia for the prevention of cardiovascular disease in the adult. Can J Cardiol.

[14] Catapano AL, Graham I, De Backer G, Wiklund O, Chapman MJ, Drexel H, Hoes AW, Jennings CS, Landmesser U, Pedersen TR, et al: 2016 ESC/EAS guidelines for the Management of Dyslipidaemias the Task Force for the Management of Dyslipidaemias of the European Society of Cardiology (ESC) and European atherosclerosis society (EAS) developed with the special contribution of the European Assocciation for Cardiovascular Prevention & Rehabilitation (EACPR). Atherosclerosis 2016, 253:281–344. 10.1016/j.atherosclerosis.2016.08.018 27594540

[15] Adhyaru BB, Jacobson TA (2016). New cholesterol guidelines for the Management of Atherosclerotic Cardiovascular Disease Risk a Comparison of the 2013 American College of Cardiology/American Heart Association cholesterol guidelines with the 2014 National Lipid Association Recommendations for patient-centered Management of Dyslipidemia. Endocrinol Metab Clin N Am.

[16] Cziraky MJ, Watson KE, Talbert RL (2009). Targeting low HDL-cholesterol to decrease residual cardiovascular risk in the managed Care setting. J Manag Care Pharm.

[17] Contois JH, Warnick GR, Sniderman AD (2011). Reliability of low-density lipoprotein cholesterol, non-high-density lipoprotein cholesterol, and apolipoprotein B measurement. J Clin Lipidol.

[18] Ramjee V, Sperling LS, Jacobson TA (2011). Non-high-density lipoprotein cholesterol versus apolipoprotein B in cardiovascular risk stratification do the math. J Am Coll Cardiol.

[19] Enkhmaa B, Prakash N, Berglund L (2018). Non-HDL-C levels and residual cardiovascular risk: do population-specific precision approaches offer any advantages?. Atherosclerosis.

[20] Grundy SM, Arai H, Barter P, Bersot TP, Betteridge J, Carmena R, Cuevas A, Davidson MH, Genest J, Kesaniemi YA (2013). An international atherosclerosis society position paper: global recommendations for the management of dyslipidemia. Journal of Clinical Lipidology.

[21] Rabar S., Harker M., O'Flynn N., Wierzbicki A. S. (2014). Lipid modification and cardiovascular risk assessment for the primary and secondary prevention of cardiovascular disease: summary of updated NICE guidance. BMJ.

[22] Pedersen TR, Tobert JA (2004). Simvastatin: a review. Expert Opin Pharmacother.

[23] Lindgren P, Jonsson B (2009). From 4S to IDEAL: the health economics of the statin trials. Eur J Cardiovasc Prev Rehabil.

[24] Simes RJ, Marschner IC, Hunt D, Colquhoun D, Sullivan D, Stewart RAH, Hague W, Keech A, Thompson P, White H (2002). Relationship between lipid levels and clinical outcomes in the long-term intervention with pravastatin in ischemic disease (LIPID) trial - to what extent is the reduction in coronary events with pravastatin explained by on-study lipid levels?. Circulation.

[25] Cui JS, Forbes A, Kirby A, Marschner I, Simes J, Hunt D, West M, Tonkin A. Semi-parametric risk prediction models for recurrent cardiovascular events in the LIPID study. BMC Med Res Methodol. 2010;10. 10.1186/1471-2288-10-27 PMC2856584 20356409

[26] Novack V, MacFadyen J, Malhotra A, Almog Y, Glynn RJ, Ridker PM (2012). The effect of rosuvastatin on incident pneumonia: results from the JUPITER trial. Can Med Assoc J.

[27] Dugani S, Akinkuolie A, Glynn RJ, Ridker PM, Mora S. Lipoprotein subclasses, size, and statin-associated incident diabetes: an analysis from the JUPITER trial. Circulation. 2014:130.

[28] Keech A, Colquhoun D, Best J, Kirby A, Simes RJ, Hunt D, Hague W, Beller E, Arulchelvam M, Baker J (2003). Secondary prevention of cardiovascular events with long-term pravastatin in patients with diabetes or impaired fasting glucose - results from the LIPID trial. Diabetes Care.

[29] Ip CK, Jin DM, Gao JJ, Meng Z, Meng J, Tan Z, Wang JF, Geng DF (2015). Effects of add-on lipid-modifying therapy on top of background statin treatment on major cardiovascular events: a meta-analysis of randomized controlled trials. Int J Cardiol.

[30] Ho LT, Lin FJ, Tseng WK, Yin WH, Wu YW, Li YH, Yeh HI, Chen JW, Wu CC, Pat TSP (2018). On-treatment lipid profiles to predict the cardiovascular outcomes in ASCVD patients comorbid with chronic kidney disease - the multi-center T-SPARCLE registry study. J Formos Med Assoc.

[31] Folse HJ, Goswami D, Rengarajan B, Budoff M, Kahn R (2014). Clinical- and cost-effectiveness of LDL particle-guided statin therapy: a simulation study. Atherosclerosis.

[32] Lawler PR, Akinkuolie AO, Chu AY, Shah SH, Kraus WE, Craig D, Padmanabhan L, Glynn RJ, Ridker PM, Chasman DI, Mora S. Atherogenic lipoprotein determinants of cardiovascular disease and residual risk among individuals with low low-density lipoprotein cholesterol. J Am Heart Assoc. 2017;6. 10.1161/JAHA.117.005549 PMC5586287 28733430

[33] Pradhan AD, Aday AW, Rose LM, Ridker PM (2018). Residual inflammatory risk on treatment with PCSK9 inhibition and statin therapy. Circulation.

[34] Soran H, Dent R, Durrington P (2017). Evidence-based goals in LDL-C reduction. Clin Res Cardiol.

[35] Randomised trial of cholesterol lowering in 4444 patients with coronary heart disease: the Scandinavian Simvastatin Survival Study (4S). Lancet. 1994;344:1383–9. 7968073

[36] Pedersen TR, Kjekshus J, Berg K, Haghfelt T, Faergeman O, Faergeman G, Pyorala K, Miettinen T, Wilhelmsen L, Olsson AG (1994). Randomised trial of cholesterol lowering in 4444 patients with coronary heart disease: the Scandinavian simvastatin survival study (4S). Atheroscler Suppl.

[37] Iakoubova OA, Tong CH, Rowland CM, Kirchgessner TG, Young BA, Arellano AR, Shiffman D, Sabatine MS, Campos H, Packard CJ (2008). Association of the Trp719Arg polymorphism in kinesin-like protein 6 with myocardial infarction and coronary heart disease in 2 prospective trials: the CARE and WOSCOPS trials. J Am Coll Cardiol.

[38] Lee S, Cannon CP (2018). Combination lipid-lowering therapies for the prevention of recurrent cardiovascular events. Curr Cardiol Rep.

[39] Ballantyne CM (2003). Current and future aims of lipid-lowering therapy: changing paradigms and lessons from the heart protection study on standards of efficacy and safety. Am J Cardiol.

[40] Farmer JA, Gotto AM (2003). The heart protection study: expanding the boundaries for high-risk coronary disease prevention. Am J Cardiol.

[41] Parish S, Offer A, Clarke R, Hopewell JC, Hill MR, Otvos JD, Armitage J, Collins R (2012). Heart protection study collaborative G: lipids and lipoproteins and risk of different vascular events in the MRC/BHF heart protection study. Circulation.

[42] Baigent C, Keech A, Kearney PM, Blackwell L, Buck G, Pollicino C, Kirby A, Sourjina T, Peto R, Collins R (2005). Efficacy and safety of cholesterol-lowering treatment: prospective meta-analysis of data from 90,056 participants in 14 randomised trials of statins. Lancet.

[43] Miller M, Cannon CP, Murphy SA, Qin J, Ray KK, Braunwald E, Investigators PI-T (2008). Impact of triglyceride levels beyond low-density lipoprotein cholesterol after acute coronary syndrome in the PROVE IT-TIMI 22 trial. J Am Coll Cardiol.

[44] Steen DL, Umez-Eronini AA, Guo J, Khan N, Cannon CP (2018). The effect of fasting status on lipids, lipoproteins, and inflammatory biomarkers assessed after hospitalization for an acute coronary syndrome: insights from PROVE IT-TIMI 22. Clin Cardiol.

[45] Wilson SR, Sabatine MS, Wiviott SD, Ray KK, De Lemos JA, Zhou S, Rifai N, Cannon CP, Morrow DA, Group TS (2011). Assessment of adiponectin and the risk of recurrent cardiovascular events in patients presenting with an acute coronary syndrome: observations from the pravastatin or atorVastatin evaluation and infection trial-thrombolysis in myocardial infarction 22 (PROVE IT-TIMI 22). Am Heart J.

[46] Bohula EA, Wiviott SD, Giugliano RP, Blazing MA, Park JG, Murphy SA, White JA, Mach F, Van de Werf F, Dalby AJ (2017). Prevention of stroke with the addition of ezetimibe to statin therapy in patients with acute coronary syndrome in IMPROVE-IT (Improved reduction of outcomes: Vytorin efficacy international trial). Circulation.

[47] Jarcho JA, Keaney JF (2015). Proof that lower is better--LDL cholesterol and IMPROVE-IT. N Engl J Med.

[48] Kato ET, Cannon CP, Blazing MA, Bohula E, Guneri S, White JA, Murphy SA, Park JG, Braunwald E, Giugliano RP. Efficacy and safety of adding ezetimibe to statin therapy among women and men: insight from IMPROVE-IT (Improved reduction of outcomes: Vytorin efficacy international trial). J Am Heart Assoc. 2017;6. 10.1161/JAHA.117.006901 PMC5721774 29151034

[49] Nichols GA, Philip S, Reynolds K, Granowitz CB, Fazio S. Increased residual cardiovascular risk in patients with diabetes and high versus normal triglycerides despite statin-controlled LDL cholesterol. Diabetes Obes Metab. 2018. 10.1111/dom.13537 PMC6587847 30225881

[50] Cui Y, Blumenthal RS, Flaws JA, Whiteman MK, Langenberg P, Bachorik PS, Bush TL (2001). Non-high-density lipoprotein cholesterol level as a predictor of cardiovascular disease mortality. Arch Intern Med.

[51] Pischon T, Girman CJ, Sacks FM, Rifai N, Stampfer MJ, Rimm EB (2005). Non-high-density lipoprotein cholesterol and apolipoprotein B in the prediction of coronary heart disease in men. Circulation.

[52] Mora Samia, Ridker Paul M. (2006). Justification for the Use of Statins in Primary Prevention: An Intervention Trial Evaluating Rosuvastatin (JUPITER)—Can C-Reactive Protein Be Used to Target Statin Therapy in Primary Prevention?. The American Journal of Cardiology.

[53] Boekholdt SM, Arsenault BJ, Mora S, Pedersen TR, LaRosa JC, Nestel PJ, Simes RJ, Durrington P, Hitman GA, Welch KMA (2012). Association of LDL cholesterol, non-HDL cholesterol, and apolipoprotein B levels with risk of cardiovascular events among patients treated with statins a meta-analysis. Jama-Journal of the American Medical Association.

[54] Sondermeijer BM, Rana JS, Arsenault BJ, Shah PK, Kastelein JJ, Wareham NJ, Boekholdt SM, Khaw KT (2013). Non-HDL cholesterol vs. apo B for risk of coronary heart disease in healthy individuals: the EPIC-Norfolk prospective population study. Eur J Clin Investig.

[55] Wongcharoen W, Sutthiwutthichai S, Gunaparn S, Phrommintikul A (2017). Is non-HDL-cholesterol a better predictor of long-term outcome in patients after acute myocardial infarction compared to LDL-cholesterol? : A retrospective study. BMC Cardiovasc Disord.

[56] de Oliveira Alvim R, Mourao-Junior CA, Magalhaes GL, de Oliveira CM, Krieger JE, Mill JG, Pereira AC (2017). Non-HDL cholesterol is a good predictor of the risk of increased arterial stiffness in postmenopausal women in an urban Brazilian population. Clinics (Sao Paulo).

[57] Rana JS, Liu JY, Moffet HH, Boklage SH, Khan I, Karter AJ (2018). Risk of incident atherosclerotic cardiovascular DiseaseEvents by achieved Atherogenic lipid levels Among62,428 statin-treated individuals with diabetes mellitus. Am J Cardiol.

[58] Cao Y, Yan L, Guo N, Yu N, Wang Y, Cao X, Yang S, Lv F (2018). Non-high-density lipoprotein cholesterol and risk of cardiovascular disease in the general population and patients with type 2 diabetes: a systematic review and meta-analysis. Diabetes Res Clin Pract.

[59] Kastelein JJ, van der Steeg WA, Holme I, Gaffney M, Cater NB, Barter P, Deedwania P, Olsson AG, Boekholdt SM, Demicco DA (2008). Lipids, apolipoproteins, and their ratios in relation to cardiovascular events with statin treatment. Circulation.

[60] Robinson JG, Wang S, Smith BJ, Jacobson TA (2009). Meta-analysis of the relationship between non-high-density lipoprotein cholesterol reduction and coronary heart disease risk. J Am Coll Cardiol.

[61] Lee EY, Yang Y, Kim HS, Cho JH, Yoon KH, Chung WS, Lee SH, Chang K (2018). Effect of visit-to-visit LDL-, HDL-, and non-HDL-cholesterol variability on mortality and cardiovascular outcomes after percutaneous coronary intervention. Atherosclerosis.

[62] Khaloo P, Hasheminia M, Tohidi M, Abdi H, Mansournia MA, Azizi F, Hadaegh F (2018). Impact of 3-year changes in lipid parameters and their ratios on incident type 2 diabetes: Tehran lipid and glucose study. Nutr Metab (Lond).

[63] Blaha MJ, Blumenthal RS, Brinton EA, Jacobson TA (2008). Non-HDL NLAT: The importance of non-HDL cholesterol reporting in lipid management. Journal of Clinical Lipidology.

[64] Degoma EM, Davis MD, Dunbar RL, Mohler ER, Greenland P, French B (2013). Discordance between non-HDL-cholesterol and LDL-particle measurements: results from the multi-ethnic study of atherosclerosis. Atherosclerosis.

[65] Elshazly MB, Quispe R, Michos ED, Sniderman AD, Toth PP, Banach M, Kulkarni KR, Coresh J, Blumenthal RS, Jones SR, Martin SS (2015). Patient-level discordance in population percentiles of the Total cholesterol to high-density lipoprotein cholesterol ratio in comparison with low-density lipoprotein cholesterol and non-high-density lipoprotein cholesterol: the very large database of lipids study (VLDL-2B). Circulation.

[66] Anderson TJ, Gregoire J, Hegele RA, Couture P, Mancini GB, McPherson R, Francis GA, Poirier P, Lau DC, Grover S (2013). 2012 update of the Canadian cardiovascular society guidelines for the diagnosis and treatment of dyslipidemia for the prevention of cardiovascular disease in the adult. Can J Cardiol.

[67] Contois JH, McConnell JP, Sethi AA, Csako G, Devaraj S, Hoefner DM, Warnick GR, Lipoproteins A (2009). Vascular diseases division working group on Best P: apolipoprotein B and cardiovascular disease risk: position statement from the AACC Lipoproteins and vascular diseases division working group on Best practices. Clin Chem.

[68] Genest J, McPherson R, Frohlich J, Anderson T, Campbell N, Carpentier A, Couture P, Dufour R, Fodor G, Francis GA (2009). 2009 Canadian cardiovascular society/Canadian guidelines for the diagnosis and treatment of dyslipidemia and prevention of cardiovascular disease in the adult - 2009 recommendations. Can J Cardiol.

[69] Authors/Task Force M, Catapano AL, Graham I, De Backer G, Wiklund O, Chapman MJ, Drexel H, Hoes AW, Jennings CS, Landmesser U, et al. 2016 ESC/EAS guidelines for the Management of Dyslipidaemias: the task Force for the Management of Dyslipidaemias of the European Society of Cardiology (ESC) and European atherosclerosis society (EAS) developed with the special contribution of the European Assocciation for Cardiovascular Prevention & Rehabilitation (EACPR). Atherosclerosis. 2016(253):281–344. 10.1016/j.atherosclerosis.2016.08.018 27594540

[70] Quispe R, Manalac RJ, Faridi KF, Blaha MJ, Toth PP, Kulkarni KR, Nasir K, Virani SS, Banach M, Blumenthal RS (2015). Relationship of the triglyceride to high-density lipoprotein cholesterol (TG/HDL-C) ratio to the remainder of the lipid profile: the very large database of Lipids-4 (VLDL-4) study. Atherosclerosis.

[71] Al-Hashmi K, Al-Zakwani I, Al Mahmeed W, Arafah M, Al-Hinai AT, Shehab A, Al Tamimi O, Al Awadhi M, Al Herz S, Al Anazi F (2016). Non-high-density lipoprotein cholesterol target achievement in patients on lipid-lowering drugs and stratified by triglyceride levels in the Arabian gulf. J Clin Lipidol.

[72] Pongsuthana S, Tivatunsakul N (2016). Optimal fasting time before measurement of serum triglyceride levels in healthy volunteers. J Med Assoc Thail.

[73] Yue F, Zhang G, Tang R, Zhang Z, Teng L, Zhang Z (2016). Age- and sex-related changes in fasting plasma glucose and lipoprotein in cynomolgus monkeys. Lipids Health Dis.

[74] Ballantyne CM, Pitt B, Loscalzo J, Cain VA, Raichlen JS (2013). Alteration of relation of atherogenic lipoprotein cholesterol to apolipoprotein B by intensive statin therapy in patients with acute coronary syndrome (from the limiting UNdertreatment of lipids in ACS with Rosuvastatin [LUNAR] trial). Am J Cardiol.

[75] Elshazly MB, Martin SS, Blaha MJ, Joshi PH, Toth PP, McEvoy JW, Al-Hijji MA, Kulkarni KR, Kwiterovich PO, Blumenthal RS, Jones SR (2013). Non-high-density lipoprotein cholesterol, guideline targets, and population percentiles for secondary prevention in 1.3 million adults: the VLDL-2 study (very large database of lipids). J Am Coll Cardiol.

[76] Kuwabara K, Harada S, Sugiyama D, Kurihara A, Kubota Y, Higashiyama A, Hirata T, Nishida Y, Kawasaki M, Takebayashi T, Okamura T (2016). Relationship between non-high-density lipoprotein cholesterol and low-density lipoprotein cholesterol in the general population. J Atheroscler Thromb.

[77] Brito FA, Pedrosa W, Maluf CB, dos Reis RCP, Fedeli LMG, Castilhos C, Barreto SM, Vidigal PG (2018). Non-HDL-C goals based on the distribution of population percentiles in ELSA-Brasil: is it time to change?. Atherosclerosis.

